# Supplementary figures and images for: Integrated multi-omics analysis and predictive modeling of heart failure using sepsis-related gene signature
Source: PLoS One. 2025 Jun 17;20(6):e0326212. doi: 10.1371/journal.pone.0326212 (PMC12173235; doi:10.1371/journal.pone.0326212)

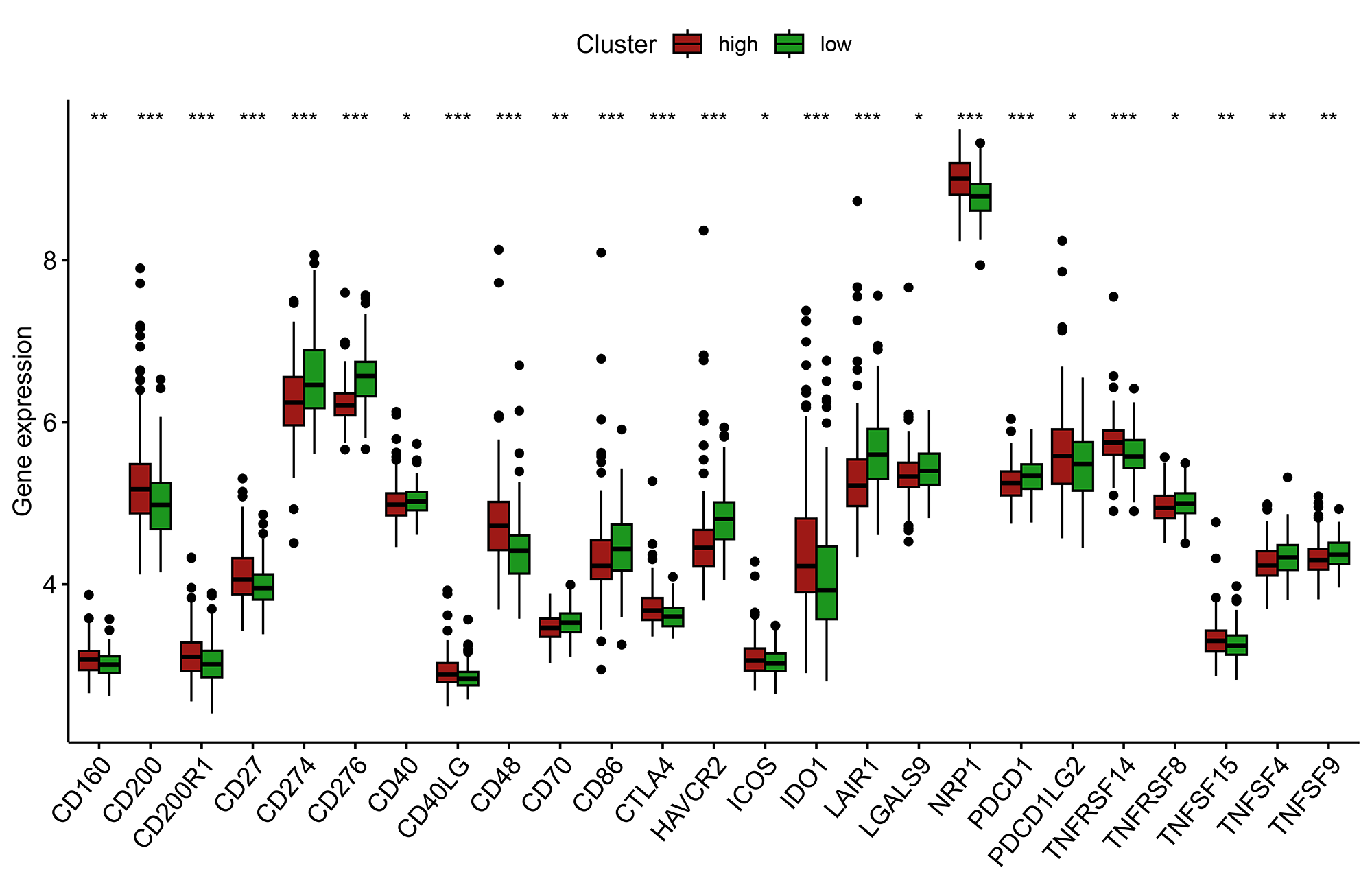

Supplement: S1 Fig — *P 0.05, **p 0.01, ***p 0.001. (TIF) [file pone.0326212.s001.tif]
