# Supplementary material for: Integrated multi-omics analysis and predictive modeling of heart failure using sepsis-related gene signature
Source: PLoS One. 2025 Jun 17;20(6):e0326212. doi: 10.1371/journal.pone.0326212 (PMC12173235; doi:10.1371/journal.pone.0326212)
Supplement: S1 Table — (DOCX) [file pone.0326212.s002.docx]

**Table S1.** Primers for qRT-PCR.

| Gene | Forward | Reverse |
| --- | --- | --- |
| GNMT | AAGAGGGCTTCAGCGTGATG | CTGGCAAGTGAGCAAAACTGT |
| SEMA4B | CAAGACGCTGTATGTGGGGG | TTGACAGTCACGCTTTGGGTC |
| FURIN | TGGTTGCTATGGGTGGTCG | CCAGAAGTGGTAATAGTCACCGA |
| RNASE2 | TGGAGCAACTTGAGTCTCGAC | CGGGGATAGGCTCTGTTATAGA |
| BEX1 | AAGCCACAAGATACCATCAGAAG | GGCGCACAGTTTTTGCCAG |
| EPHX2 | ACCACTCATGGATGAAAGCTACA | TCAGGTAGATTGGCTCCACAG |
| GAPDH | GGAGCGAGATCCCTCCAAAAT | GGCTGTTGTCATACTTCTCATGG |
