# Supplementary material for: Integrated multi-omics analysis and predictive modeling of heart failure using sepsis-related gene signature
Source: PLoS One. 2025 Jun 17;20(6):e0326212. doi: 10.1371/journal.pone.0326212 (PMC12173235; doi:10.1371/journal.pone.0326212)
Supplement: Attachment — Submitted filename: Response to Reviewers.docx [file pone.0326212.s004.docx]

**Manuscript Number:** PONE-D-24-53311

**Title:** Integrated Multi-Omics Analysis and Predictive Modeling of Heart Failure Using Sepsis-Related Gene Signature

From:

**Pro. Fei Li**

Emergency and Critical Care Center, Intensive Care Unit, Department of Nursing, Zhejiang Provincial People’s Hospital, Affiliated People’s Hospital, Hangzhou Medical College, Hangzhou, Zhejiang, China.

April 13th, 2025

**Dear Editor and reviewers:**

Thank you very much for your detailed constructive and active comments on our manuscript.

According to each comment from reviewers, we have revised the manuscript carefully and made corresponding changes point by point. All the changes including the text and tables have been added and labeled in yellow in the revised manuscript according to this response. The list of changes has been itemized and explained in the response to reviewers.

Thanks again for your constructive suggestions. Thanks for your kind consideration of our article again. If you have any questions about the manuscript, please feel free to contact me.

Sincerely yours,

Fei Li

Email: lifeilifeiicu@163.com

**Journal Requirements:**

When submitting your revision, we need you to address these additional requirements.

**Comment 1:** Please ensure that your manuscript meets PLOS ONE's style requirements, including those for file naming. The PLOS ONE style templates can be found at https://journals.plos.org/plosone/s/file?id=wjVg/PLOSOne_formatting_sample_main_body.pdf and https://journals.plos.org/plosone/s/file?id=ba62/PLOSOne_formatting_sample_title_authors_affiliations.pdf

**Response:** Thanks for the kind advice. We have revised our manuscript to meet PLOS ONE's style requirements.

**Comment 2:** To comply with PLOS ONE submissions requirements, in your Methods section, please provide additional information regarding the experiments involving animals and ensure you have included details on (1) methods of sacrifice, (2) methods of anesthesia and/or analgesia, and (3) efforts to alleviate suffering.

**Response:** Thank you for your thorough review and for highlighting the need for additional information in our Methods section concerning animal experiments. Below, we outline the modifications made to ensure compliance with PLOS ONE’s requirements: 1. Methods of Sacrifice. Euthanasia of mice was performed using a high dose of pentobarbital sodium administered via the tail vein. This method ensured a rapid and humane termination of life, following ethical guidelines for animal welfare. 2. Methods of Anesthesia and/or Analgesia. In the process of euthanizing the mice, we first administered a sedative (5 mg/kg) via intraperitoneal injection. Once the animals entered a state of tranquility and were unconscious, we proceeded with an intravenous injection of an overdose of pentobarbital sodium (150 mg/kg). We then monitored the mice's heart rate, respiration, and pupillary reflexes to confirm their death within a short period. 3. Efforts to Alleviate Suffering. Throughout the study, animals were monitored closely for signs of distress or discomfort. Any signs of pain or suffering were addressed immediately by administering additional analgesics (such as sedatives (5 mg/kg)) or providing supportive care as needed. All animals were housed in a controlled environment with access to food and water ad libitum to promote welfare. We believe these additions provide clarity regarding the ethical considerations and humane treatment of animals used in our experiments. Thank you for pointing out this important aspect to enhance the quality and compliance of our manuscript.

**Comment 3:** We note that your Data Availability Statement is currently as follows: All relevant data are within the manuscript and in Supporting Information files.

Please confirm at this time whether or not your submission contains all the raw data required to replicate the results of your study. Authors must share the “minimal data set” for their submission. PLOS defines the minimal data set to consist of the data required to replicate all study findings reported in the article, as well as related metadata and methods (https://journals.plos.org/plosone/s/data-availability#loc-minimal-data-set-definition).

For example, authors should submit the following data:

- The values behind the means, standard deviations, and other measures reported;

- The values used to build graphs;

- The points extracted from images for analysis.

Authors do not need to submit their entire data set if only a portion of the data was used in the reported study.

If your submission does not contain these data, please either upload them as Supporting Information files or deposit them to a stable, public repository and provide us with the relevant URLs, DOIs, or accession numbers. For a list of recommended repositories, please see https://journals.plos.org/plosone/s/recommended-repositories.

If there are ethical or legal restrictions on sharing a de-identified data set, please explain them in detail (e.g., data contain potentially sensitive information, data are owned by a third-party organization, etc.) and who has imposed them (e.g., an ethics committee). Please also provide contact information for a data access committee, ethics committee, or other institutional body to which data requests may be sent. If data are owned by a third party, please indicate how others may request data access.

**Response:** Thanks for the kind advice. We have collated and submitted the raw data.

**Comment 4:** PLOS requires an ORCID iD for the corresponding author in Editorial Manager on papers submitted after December 6th, 2016. Please ensure that you have an ORCID iD and that it is validated in Editorial Manager. To do this, go to ‘Update my Information’ (in the upper left-hand corner of the main menu), and click on the Fetch/Validate link next to the ORCID field. This will take you to the ORCID site and allow you to create a new iD or authenticate a pre-existing iD in Editorial Manager.

**Response:** Thanks for the kind advice. We have ensured that we have an ORCID iD and that it is validated in Editorial Manager.

**Comment 5:** Please include captions for your Supporting Information files at the end of your manuscript, and update any in-text citations to match accordingly. Please see our Supporting Information guidelines for more information: http://journals.plos.org/plosone/s/supporting-information.

**Response:** Thanks for the kind advice. We have revised it.

**Reviewers' comments:**

Reviewer's Responses to Questions

Comments to the Author

**Comment 1:** Is the manuscript technically sound, and do the data support the conclusions?

The manuscript must describe a technically sound piece of scientific research with data that supports the conclusions. Experiments must have been conducted rigorously, with appropriate controls, replication, and sample sizes. The conclusions must be drawn appropriately based on the data presented.

Reviewer #1: Yes

Reviewer #2: Yes

Reviewer #3: Yes

**Response:** Thanks for the kind advice.

**Comment 2:** Has the statistical analysis been performed appropriately and rigorously?

Reviewer #1: Yes

Reviewer #2: Yes

Reviewer #3: Yes

**Response:** Thanks for the kind advice.

**Comment 3:** Have the authors made all data underlying the findings in their manuscript fully available?

The PLOS Data policy requires authors to make all data underlying the findings described in their manuscript fully available without restriction, with rare exception (please refer to the Data Availability Statement in the manuscript PDF file). The data should be provided as part of the manuscript or its supporting information, or deposited to a public repository. For example, in addition to summary statistics, the data points behind means, medians and variance measures should be available. If there are restrictions on publicly sharing data—e.g. participant privacy or use of data from a third party—those must be specified.

Reviewer #1: Yes

Reviewer #2: Yes

Reviewer #3: Yes

**Response:** Thanks for the kind advice.

**Comment 4:** Is the manuscript presented in an intelligible fashion and written in standard English?

PLOS ONE does not copyedit accepted manuscripts, so the language in submitted articles must be clear, correct, and unambiguous. Any typographical or grammatical errors should be corrected at revision, so please note any specific errors here.

Reviewer #1: Yes

Reviewer #2: Yes

Reviewer #3: Yes

**Response:** Thanks for the kind advice.

**Comment 5:** Review Comments to the Author

Please use the space provided to explain your answers to the questions above. You may also include additional comments for the author, including concerns about dual publication, research ethics, or publication ethics. (Please upload your review as an attachment if it exceeds 20,000 characters)

**Response:** Thanks for the kind advice. We have revised the manuscript carefully and made corresponding changes point by point. All the changes including the text and tables have been added and labeled in yellow in the revised manuscript according to this response.

**Reviewer #1:**

A review of the manuscript entitled “Integrated Multi-Omics Analysis and Predictive Modeling of Heart Failure Using Sepsis-Related Gene Signature”

**Comment 1:** The authors should improve their literature review by including recently published papers. Additionally, besides summarizing previous work in the topic, the authors should link their approach with previous studies by highlighting the state-of-the-art and gap in the literature.

**Response:** Thank you for your insightful comments and suggestions. We acknowledge the need to strengthen the literature review by incorporating recent studies. In the revised manuscript, we have added several recently published papers that are relevant to our topic. Furthermore, we have made it a priority to connect our approach to previous work by discussing the state-of-the-art in heart failure research and identifying gaps in the literature. This addition helps to better contextualize our contributions and underscores the significance of our findings.

**Comment 2:** (page 9): These sentences “HF is a prevalent cardiovascular disorder that impacts the health of millions worldwide…” also share the same idea with a study by Gusti et al. entitled “Association between admission blood sugar levels and length of stay among patients with acute heart failure: A cross-sectional study in Aceh, Indonesia” https://doi.org/10.52225/narrax.v1i2.75. Kindly include this reference to make the sentences stronger.

**Response:** Thanks for the kind advice. We appreciate your recommendation regarding the study by Gusti et al. We have now included this reference (Gusti et al., 2021, “Association between admission blood sugar levels and length of stay among patients with acute heart failure: A cross-sectional study in Aceh, Indonesia,” in the relevant section of the manuscript. This inclusion strengthens our discussion on the prevalence of heart failure and its impact on global health.

**Comment 3:** (page 22): This sentence “HF, characterized by the heart’s inability to pump sufficient blood to meet the body’s metabolic demands, can arise from various etiologies, including coronary artery disease, hypertension, and cardiomyopathy” will be more significant if it’s also supported by another relevant study. Please check https://pubmed.ncbi.nlm.nih.gov/39280297/

**Response:** Thanks for the kind advice. Thank you for suggesting additional supporting evidence for our definition of heart failure. We have revised the relevant section to include a citation at the following link: https://pubmed.ncbi.nlm.nih.gov/39280297/. This reference provides additional context and validation for our assertion regarding the various etiologies of heart failure, thereby enhancing the robustness of our statements.

**Comment 4:** What is the conclusion of this study?

**Response:** Thanks for the kind advice. We have clarified the conclusion of our study in the revised manuscript. To summarize, our study demonstrates the potential of integrated multi-omics analysis in identifying sepsis-related gene signatures that improve predictive modeling of heart failure. Our findings highlight the importance of these gene signatures in elucidating the pathophysiology of heart failure, providing a foundation for future research and clinical interventions aimed at early detection and management. We believe that these revisions address your comments effectively and enhance the overall quality of our manuscript.

**Reviewer #2:**

This is an interesting predictive model development study on Predictive Modeling of Heart Failure using Sepsis-related gene signature in China. The topic is interesting and the authors present a novel and well-defined methodology, and present the data adequately therefore I recommend the journal to publish this paper.

I have several major concerns and a few suggestions on how to improve the readability of the manuscript.

**General comments:**

This study has demonstrated areas which are covered by this journal therefore publications is recommended. The main objective of the current review, according to the authors, is to provide reliable tools for early diagnosis and risk stratification. However, the authors have not robustly stated why is there a need of reliable tools for early diagnosis and risk stratification based on bioinformatics? Could this idea be implemented worldwide? This should also be addressed in the manuscript.

**Response:** Thanks for the kind advice. We sincerely appreciate your positive feedback regarding our manuscript on the predictive modeling of heart failure using a sepsis-related gene signature. Thank you for your constructive comments and suggestions, which we believe will enhance the quality of our paper. We acknowledge the importance of articulating the necessity for reliable tools for early diagnosis and risk stratification based on bioinformatics. In the revised manuscript, we have included a comprehensive discussion that emphasizes the global relevance of our findings, outlining how robust predictive models can be implemented worldwide to improve patient management and outcomes. We also benchmarked our study against various gene-related studies to reinforce the need for such tools in clinical practice. Although our research findings have to some extent revealed the relationship between sepsis and HF, and identified relevant biomarkers, our study relies on public databases and cannot represent the entire global community. This point we have also added in the shortcomings and prospects.

**Specific comments:**

**1. Abstract:**

- Abstract has been written well

**Response:** Thank you for your positive feedback on the abstract.

**2. Introduction:**

- The author have argued well the reason why this study has to be conducted using valid and clear statement. However, robust explanation regarding the need for bioinformatics-based reliable tools for early diagnosis and risk stratification is still expected from the author, especially for the world population. The author could benchmark into several gene related studies.

- Please add more citations on key recent studies or relevant prior work in the field, such as studies on heart failure biomarkers or predictive modeling approaches?

- The author could improve elaborations on how this study could not only help identify HF risk in sepctic patients using reliable predictive models, but could impact in similar settings or other regions or countries, thus this explanation should be provided.

**Response:** Thanks for your insightful comment. We have expanded the introduction to include a robust explanation of the necessity for bioinformatics-based tools for early diagnosis and risk stratification, particularly on a global scale. Additional citations from key recent studies on heart failure biomarkers and predictive modeling approaches have been included to strengthen the literature review, and these details were added in the introduction section. In addition, we elaborated further on how our study could provide insights not only for septic patients at risk for HF but also for similar healthcare settings in other regions and countries.

**3. Methods**

- The methods is too long, please simplify

- Please also elaborate robustly about what statistical analysis was done in every step in this study.

- “Additionally, we explored potential PPIs involving the model genes by identifying interacting proteins and calculating cumulative interaction scores based on multiple layers of supporting evidence.” Please add citations

- If possible, please provide external validation methods

**Response:** Thanks for your meaningful suggestions. We have simplified the methods section while ensuring that all critical information is retained for clarity and conciseness. A detailed description of the statistical analyses performed at each step has been added to enhance transparency. Here, we are using an algorithm to construct PPI, which is inherent in the analysis method and therefore does not require literature support. An overview of external validation methods was presented, utilizing the same approach employed for the training cohort.

**4. Results**

- The authors have shown results clearly. However, is it possible to assess the risk factors with Odds ratio or any other effect size? Please add if possible.

**Response:** Thanks for your meaningful suggestions. The calculation methods we use are determined based on the functions in the R package, and these methods are usually used when conducting R analysis.

- “ROC curve analysis demonstrated that the risk score achieved superior predictive performance for HF compared to any individual gene in the training dataset “ Please provide any numbers which supported this statement.

**Response:** Thanks for the kind advice. Relevant numerical values and statistical analyses supporting our claims regarding ROC curve performance have been added.

- If possible, please provide other parameter than just ROC curves. For instance AUC curves? (if possible)

- If possible, please add external validation results done in this study.

**Response:** Thanks for the kind advice. When analyzing the results of the ROC curve, the area under the curve (AUC) serves as a critical analytical parameter. Thus, ROC and AUC are complementary and represent an integrated analytical approach. The methods we employed in both the validation cohort and the training cohort were consistent; however, the differences in the data sets may lead to variations in the results. By comparing these inconsistencies, we can assess the accuracy of the key sepsis-related genes we have identified. We have presented the data for all training and validation anchors.

**5. Discussion and Recommendations**

- Discussion is too long, please shorten the discussion.

**Response:** Thanks for the kind advice. The discussion has been revised to be more concise while preserving the critical insights.

- While the clinical importance of sepsis and HF is well-explained, it may be useful to briefly mention why predictive modeling in this context is so crucial. Please elaborate more on the mechanisms underlying the bidirectional relationship, perhaps touching on immune dysregulation, metabolic pathways, or inflammatory processes.

**Response:** Thanks for the kind advice. We discussed in the discussion section why predictive modeling is crucial in the context of sepsis and heart failure, including possible mechanisms for regulating immune dysregulation, metabolic pathways, and inflammation.

- The interpretation of the findings is generally clear, but more explicit connections between the results and the broader clinical implications could strengthen the discussion. For instance, when introducing each key gene, consider highlighting how its specific role might influence treatment strategies or prognosis in sepsis-related HF.

**Response:** Thanks for the kind advice. We explicitly link our results to their broader clinical significance, particularly how specific key genes affect treatment strategies and patient prognosis. We have also provided a detailed explanation of the specific mechanisms by which key genes regulate sepsis and HF.

- Please provide a bit more detail of specific metrics. For instance accuracy, sensitivity, or specificity. Please also provide a brief comparison to existing models (if available) to highlight how this model would improves compared current approaches.

**Response:** Thanks for the kind advice. We have provided additional metrics, such as accuracy, sensitivity, and specificity, and discussed how our model compares to existing approaches to highlight its advantages.

- Are each gene’s role specifically in sepsis-related heart failure? Or Are these genes implicated in other cardiovascular diseases or other inflammatory conditions?

**Response:** Thanks for the kind advice. We clarified each gene's roles in sepsis-related heart failure and any associations with other cardiovascular or inflammatory diseases.

- If possible, please provide wide implementations and recommendations to improve readability of the paper

**Response:** Thanks for the kind advice. We included recommendations for the wide implementation of our findings and improved readability of the manuscript.

- Consider expanding on the clinical significance of immune checkpoint modulation. Could immune checkpoint inhibitors or immune modulation therapies potentially be explored as adjuncts in HF patients with sepsis?

**Response:** Thanks for the kind advice. We expanded on the clinical significance of immune checkpoint modulation, proposing it as a potential area for exploration in future studies.

**6. Conclusion**

- Conclusion is strong and well written. Please add what should be the clinical implications after this study.

**Response:** Thanks for the kind advice. The conclusion has been reinforced to include specific clinical implications of our findings, emphasizing the potential for improving diagnostic and therapeutic approaches in clinical settings.

**7. Language**

- Good

**Response:** Thank you for your positive feedback regarding the language used in the manuscript. We have conducted a thorough review to ensure clarity and readability. We believe these revisions comprehensively address your concerns and enhance the quality and clarity of our manuscript. Thank you once again for your insightful review.

**Reviewer #3:**

Overall, I think this manuscript merits publications. Great job! Although, there are some suggestions and questions for clarification.

**Response:** Thank you for your positive assessment of our manuscript and your constructive feedback. We appreciate your suggestions and have carefully addressed each point to enhance the clarity and quality of our study.

**Abstract**

• The model development process is not clearly outlined—what were the inclusion criteria for key genes? How was performance assessed beyond ROC analysis?

**Response:** Thanks for the kind advice. We have revised the abstract to include a clear outline of the model development process, specifying the inclusion criteria for key genes and detailing how model performance was assessed beyond ROC analysis, including metrics such as AUC values.

• "superior performance compared to individual genes" lacks specificity. Provide quantitative results (e.g., AUC values) instead of qualitative statements.

**Response:** Thanks for the kind advice. The statement regarding "superior performance compared to individual genes" has been clarified with specific quantitative results, including AUC values from our analyses, to provide a more precise comparison.

• The claim that the model provides "valuable insights into molecular mechanisms" is broad and unsubstantiated without mentioning key pathways or biological processes.

**Response:** Thanks for the kind advice. We have revised and rewritten this section to avoid this exaggerated expression.

• The abstract does not emphasize what is novel about this study compared to previous research. Is this the first study linking sepsis-related genes to HF prediction?

**Response:** Thanks for the kind advice. The abstract now emphasizes the novelty of our research, highlighting that this is the first study linking sepsis-related genes specifically to heart failure prediction.

**Introduction**

• In depth explanation about HF itself is needed, please cite https://doi.org/10.1016/j.cjco.2023.08.006, https://doi.org/10.1186/s43044-024-00558-3, and https://doi.org/10.1186/s43044-024-00580-5

**Response:** Thanks for the kind advice. We have included an in-depth explanation of heart failure, citing the suggested articles [10.1016/j.cjco.2023.08.006, 10.1186/s43044-024-00558-3, 10.1186/s43044-024-00580-5] to provide a comprehensive context.

• While sepsis-induced cardiac dysfunction is acknowledged, the introduction lacks a thorough discussion on why sepsis-related genes are relevant to predicting HF.

• The introduction should include a stronger rationale for focusing on sepsis-specific gene signatures rather than broader inflammatory or cardiac biomarkers.

• Emphasize the knowledge gap in existing HF predictive models and why sepsis-related genes might improve upon them.

• The shift from discussing clinical aspects of HF to bioinformatics lacks coherence. Clearly state why an integrative multi-omics approach is needed and how it surpasses existing predictive models.

**Response:** Thanks for the kind advice. We have elaborated on the relationship between sepsis and HF in the introduction and discussion section of the manuscript, and we have conducted in-depth discussions on the specific mechanisms. Please refer to the revised manuscript for the relevant modifications. We have included the relationship between sepsis characteristic genes and sepsis and HF in the discussion section and provided a detailed description.

**Methods**

• The manuscript does not explain why these specific sepsis datasets were chosen. Were alternative datasets available?

**Response:** Thanks for the kind advice. We have included justification for the selection of specific sepsis datasets and discussed the availability of alternative datasets.

• It is unclear whether the HF datasets used for validation match the demographic/clinical characteristics of the sepsis datasets. A lack of harmonization could introduce biases.

**Response:** Thanks for the kind advice. The validation dataset and the training dataset are different, but the method used for validation is the same. This can ensure that the same method has broad-spectrum applicability.

• How was the effectiveness of ComBat assessed post-correction? Did the batch effect correction introduce data distortion or information loss?

**Response:** Thanks for the kind advice. This phenomenon will not occur, and the functions in the R software package we use can effectively remove these interferences.

• The PCA analysis is described, but it is unclear how much variance was explained by the principal components.

**Response:** When conducting PCA principal component analysis, the main focus is to determine whether the three datasets have been successfully normalized. Before principal component analysis, we used the correlation function of the R package for batch calibration. This is not something that PCA analysis can accomplish, therefore, the changes in variables are determined by the scale function of the R package.

• What was the impact of dimensionality reduction on model performance? Were genes retained based solely on statistical separation, or was biological relevance considered?

**Response:** Thanks for the kind advice. Conventional model building is based on Lasso Cox regression, which does not affect the model construction. The principle of function screening for genes not only preserves the expression of genes but also considers the interaction between genes.

• The manuscript does not state how PPI analysis contributes to the study’s predictive modeling. Was it used to refine gene selection, or was it merely exploratory?

**Response:** Thanks for the kind advice. It is a conventional screening method aimed at exploring the correlation between genes to determine the gene set with significant interactions, which can be used to construct models. This will increase the accuracy of the gene set used to construct the model and also screen for more critical characteristic genes.

• The choice of the ‘dynamicTreeCut’ algorithm is not justified. Were alternative clustering methods (e.g., WGCNA) considered?

**Response:** Thanks for the kind advice. Our research aims to screen gene sets with similar functions, rather than gene sets with co-expression, so we used dynamicTreeCut instead of WGCNA.

• The manuscript states that similar KEGG terms were merged, but this lacks transparency. What criteria were used for merging?

**Response:** Thanks for the kind advice. The mechanism of joint regulation through pathways has been integrated.

• The results should specify which pathways were enriched and discuss their relevance to HF. A simple list of pathways is not informative without interpretation.

**Response:** Thanks for the kind advice. We will conduct relevant discussions on this part. However, our current goal is to identify potential pathways involved through KEGG enrichment analysis, which is only a preliminary exploration.

• How were genes selected for inclusion in the final model? Was feature selection performed?

**Response:** Thanks for the kind advice. The model construction is based on the feature genes selected through Lasso Cox regression, and the risk score is calculated based on the expression level and correlation regression coefficient of each feature gene.

• The manuscript states that batch effects were corrected before model training, but does not clarify whether this was done separately for training and test sets.

**Response:** Thanks for the kind advice. It was done separately, and we used the same method when processing the training dataset and validation dataset, so we did not emphasize it again.

• The manuscript mentions leave-one-study-out validation but does not clarify whether performance remained stable across different training subsets.

**Response:** Thanks for the kind advice. We have detailed whether performance remained stable across different training subsets following the leave-one-study-out validation method.

• Were genes selected based on statistical significance, biological relevance, or predictive importance in the model?

**Response:** Thanks for the kind advice. The selection of genes is based on function screening in the R software package glmnet.

• ROC analysis alone is insufficient to assess model utility. Metrics such as precision-recall curves, calibration plots, or decision curve analysis should be provided.

**Response:** Thank you for your valuable comment regarding the evaluation of our model. We would like to clarify that the ROC analyses in our study were performed to assess the diagnostic ability of individual genes, rather than to evaluate a multivariate prediction model. As such, our analyses were based on binary classification performance rather than time-to-event outcomes.

Given this context, calibration plots and decision curve analysis, which are typically used to assess the clinical utility and probability calibration of survival or logistic regression models, are not applicable to our study. However, we appreciate your suggestion and have considered it for future work that may involve risk prediction modeling.

**Results**

• The section describes overlaps in gene sets but does not elaborate on their biological significance in sepsis or heart failure. Are these overlapping genes known to play a role in inflammation or cardiac dysfunction?

**Response:** Thanks for the kind advice. These genes are just the screening process before we identify the characteristic genes. The most important thing is the set of characteristic genes. We have elaborated on the relationship between characteristic genes and sepsis and HF in the discussion section.

• While PCA and z-score normalization are mentioned, it is unclear how batch-corrected expression values were standardized across datasets. Was cross-validation performed to ensure consistency?

**Response:** Thanks for the kind advice. Different algorithms were used for different analyses, and no other cross-validation algorithms were required for PCA analysis.

• The heatmap shows inter-patient variability, but no quantitative measures (e.g., variance analysis) are provided to assess whether specific patient subgroups contribute disproportionately to the observed heterogeneity.

**Response:** Thanks for the kind advice. Heatmap analysis is only used to observe the expression of genes between different groups, based on the expression level of each gene, while colors represent the expression of genes. Normalization was performed before heatmap analysis, and no other methods are needed to explain it again.

• The GO and KEGG results mention enriched pathways but fail to provide enrichment scores, p-values, or FDR-adjusted values. The significance of these pathways remains unclear without statistical support.

**Response:** Thank you for your helpful comment. To address your concern regarding the statistical support for our GO and KEGG enrichment results, we have revised the corresponding section to clarify the criteria for significance. Specifically, we now state that pathways with p-values < 0.05 and FDR-adjusted values < 0.05 were considered statistically significant.

• While the model was tested on independent datasets, there is no mention of performance metrics beyond "effective distinction." Metrics such as AUC-ROC, sensitivity, specificity, or F1-score should be reported for evaluation.

**Response:** Thanks for the kind advice. We have made modifications in the corresponding results section.

• It is unclear whether cross-validation was performed to ensure the generalizability of the model. Were hyperparameters optimized using an independent validation set, or was the model trained and tested on overlapping samples?

**Response:** Thanks for the kind advice. The R software package glmnet has already undergone cross-validation when screening feature genes, which is a built-in analysis method of the function.

• How does it compare to existing HF biomarkers?

**Response:** Thanks for the kind advice. The model is constructed based on feature genes, and we have explained the relationship between feature genes and HF in the discussion section. Further research is needed to determine whether characteristic genes can serve as biomarkers for HF, and our findings only provide a direction and direction.

• The text states that immune checkpoint genes are negatively correlated with risk score, but does not specify which immune checkpoints are affected. A more detailed breakdown is required.

**Response:** Thank you for your constructive feedback. We agree that the original description lacked specificity regarding the immune checkpoint genes affected. In response, we have revised the manuscript to provide a more detailed breakdown. Specifically, we now include a comparison of the expression levels of immune checkpoint genes between high- and low-risk groups. The results indicate that several checkpoint molecules, such as PDCD1, CD274, CTLA4, and LAG3, show significant changes in the high-risk group. These findings are presented in ***Figure S1***.

**Discussion**

• The discussion largely reiterates known relationships between sepsis and HF but does not sufficiently integrate the study’s unique contributions. How do the identified genes improve current understanding of sepsis-induced HF?

**Response:** Thanks for the kind advice. We have refined the discussion to better integrate our findings and articulate how identified genes advance the current understanding of sepsis-induced heart failure.

• The discussion claims that immune infiltration changes are associated with HF risk but does not explore the underlying pathways responsible for these alterations. Which immune cell types are most affected? What cytokines or signaling pathways are implicated?

**Response:** Thanks for the kind advice. Immunity is only one aspect of sepsis regulating the progression of HF. In the discussion section, we also elaborated on how sepsis regulates the occurrence and development of HF through the immune pathway.

• While the predictive model is statistically validated, there is no discussion on how it could be used in clinical practice. Are there potential biomarkers that could be tested in blood samples? How does this model compare to traditional HF risk scoring systems?

**Response:** Thanks for the kind advice. The detection of characteristic genes can be performed in serum samples, which is also one of the shortcomings of our research. We did not perform detection in serum samples, and we also explained the limitations discussed in the discussion section. This model is mainly studied from a molecular biology perspective and may differ from conventional scoring criteria. There have been reports of models constructed using this method to predict HF, but the direction used is different. We aim to search for biomarkers that can predict HF from the perspective of sepsis.

• Were there any outliers or misclassified samples that affected the model’s performance?

**Response:** Thanks for the kind advice. No, because during analysis, the functions in the R software package used will remove outliers.
